# Intraventricular Pilocytic Astrocytoma in a 75-Year-Old Adult Patient: A Case Report

**DOI:** 10.7759/cureus.79589

**Published:** 2025-02-24

**Authors:** Naoki Wakuta, Hitoshi Tsugu, Kenichi Nishiyama, Mikiko Aoki, Hiroshi Abe

**Affiliations:** 1 Department of Neurosurgery, Fukuoka University Chikushi Hospital, Fukuoka, JPN; 2 Department of Neurosurgery, Fukuoka Red cross Hospital, Fukuoka, JPN; 3 Department of Pathology, Fukuoka Red Cross Hospital, Fukuoka, JPN; 4 Department of Pathology, Fukuoka University School of Medicine, Fukuoka, JPN; 5 Department of Neurosurgery, Faculty of Medicine, Fukuoka University, Fukuoka, JPN

**Keywords:** 75 years old patient, immunohistochemical study, intra-ventricular adult pilocytic astrocytoma (ivpas), neuroendoscopic surgery, septum pellucidum

## Abstract

Adult pilocytic astrocytoma often presents with more aggressive behavior and poorer clinical outcomes than generally appreciated as pilocytic astrocytoma. Additionally, mortality increases with age at diagnosis; even more so for intraventricular lesions. Adult intraventricular pilocytic astrocytoma is extremely rare, and the clinical characteristics and pathological information are not well-described. We report a 75-year-old woman who presented with a progressive gait disorder, cognitive function decline, and impaired consciousness. Magnetic resonance imaging revealed hydrocephalus and an intraventricular tumor extending along the medial wall of the lateral ventricle, with another isolated lesion. Endoscopic surgical resection was performed to remove the tumor at the septum pellucidum safely, and the histologic and molecular findings were consistent with pilocytic astrocytoma. This case involved unique characteristics as the oldest known patient with intraventricular pilocytic astrocytoma at the rare location of the septum pellucidum and with multiple lesions. This case may aid the diagnosis in similar cases. It is important to consider this diagnosis in the differentials for intraventricular neoplasms. Further studies to elucidate specific molecular findings and indications for appropriate treatment are required to improve patient outcomes.

## Introduction

Pilocytic astrocytomas (PA) are the most common World Health Organization (WHO) Grade I astrocytic neoplasms in children. On the other hand, the incidence in adults is only 0.8% of all central nervous system neoplasms [1]. The incidence in patients over 60 years of age was only 1.9% of all adult PA cases on the basis of epidemiological research data [2], and intraventricular PA (IVPA) is remarkably rare. Adult PA often presents with aggressive behavior and poor clinical outcomes, especially as age at diagnosis increases [1,2], and further clinical information is required to establish specific treatment indications or stratification for this subgroup. We report a pathologically diagnosed case of adult IVPA to support the diagnosis and treatment in similar cases. This article was previously presented as a meeting abstract at the Annual Meeting of the Japanese Society for Neuroendoscopy in Tokyo on 7 November 2024.

## Case presentation

**Figure 1:**
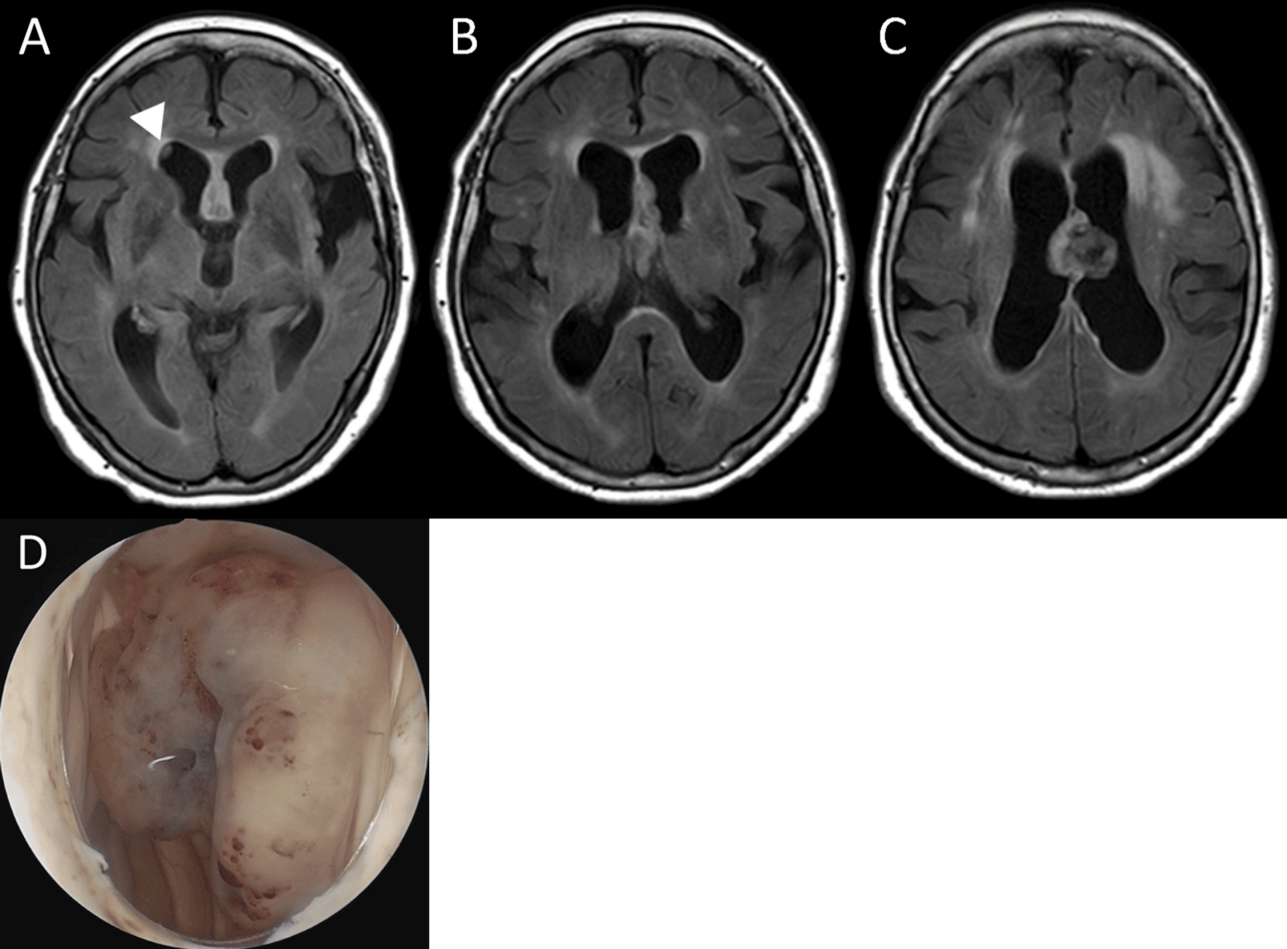
Preoperative MRI and intraoperative endoscopic view. Preoperative MRI (A–C) showing an intraventricular tumor adjacent to the septum pellucidum and extending forward along the median side of the walls of the lateral ventricles, with an isolated small lesion (arrowhead in panel A) inside the right frontal horn. Intraoperative endoscopic view (D) demonstrating a soft pink-tan-colored tumor covered by a fibrous capsule. MRI: magnetic resonance imaging.

A 75-year-old woman with Stage 4 chronic kidney disease and no other significant medical history presented with a progressive gait disorder and declining cognitive function. The functional impairment had worsened rapidly within one month, and she then developed impaired consciousness. She presented to the emergency department and was referred to our department for further management. Magnetic resonance imaging (MRI) confirmed moderate hydrocephalus and a 28-mm mixed cystic and solid mass at the septum pellucidum that extended along the medial wall of the lateral ventricle. The radiological impression was suspicious for central neurocytoma, and another isolated small lesion was detected inside the right frontal horn. They demonstrated hypointensity on T1-weighted imaging, hyperintensity on T2-weighted imaging, and iso- to hyperintensity on fluid-attenuated inversion recovery imaging, with abnormal signals suggesting calcification and a cystic lesion. Contrast-enhanced MRI was not performed because of severe renal dysfunction (Figure 1A-1C). The patient underwent endoscopic removal of the tumor. The intraoperative endoscopic view demonstrated a soft pink-tan-colored tumor covered by a fibrous capsule that was moderately vascular (Figure 1D), and the foramen of Monro was patent.

**Figure 2:**
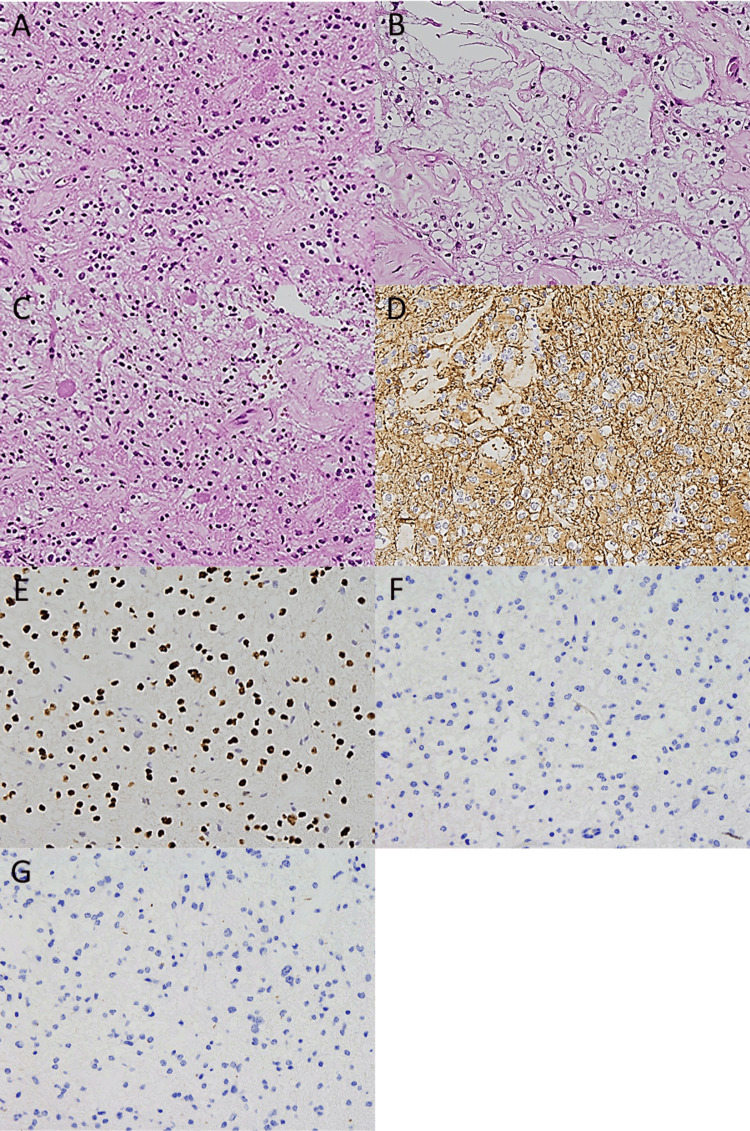
Results of pathology studies. Histopathological examination showing a biphasic appearance, with compact areas and loose and microcystic regions (A, B). Numerous eosinophilic granular bodies (C) are also observed. Immunohistochemically, the cells were positive for glial fibrillary acid protein (D) and Olig2 (E), and negative for mutation of IDH1 R132H (F) and BRAF V600E (G). A–C: H&E, ×20; D-G: immunostaining, ×20. H&E: hematoxylin and eosin

As intraoperative pathological examination suggested PA, only the lesion at the septum pellucidum was resected, in a piecemeal fashion, using bimanual instrumentation. The final pathological examination was performed with additional analysis at an outside institution. Histological examination showed a biphasic appearance composed of compacted cellular areas with bipolar cells with long, hair-like processes and loose textured microcystic areas, with smaller cells with round-to-oval nuclei dispersed by cystic changes. Eosinophilic granular bodies, Rosenthal fibers, hyaline vessels, and calcifications were present (Figure 2A-2C). Necrosis was not observed. Microvascular proliferation and anaplastic features were not detected, and mitotic figures were not evident. Immunohistochemical studies demonstrated that the neoplastic cells exhibited positivity for glial fibrillary acid protein, Olig2, and ATRX, and negativity for mutation of IDH1 R132H, BRAF V600E, and H3K27M (Figure 2D-2G). The Ki67 labeling index was 2%. Molecular testing, including KIAA1549/BRAF fusion analysis, could not be performed. The overall histologic, immunohistochemical, and molecular features were consistent with PA, WHO Grade 1.

**Figure 3:**
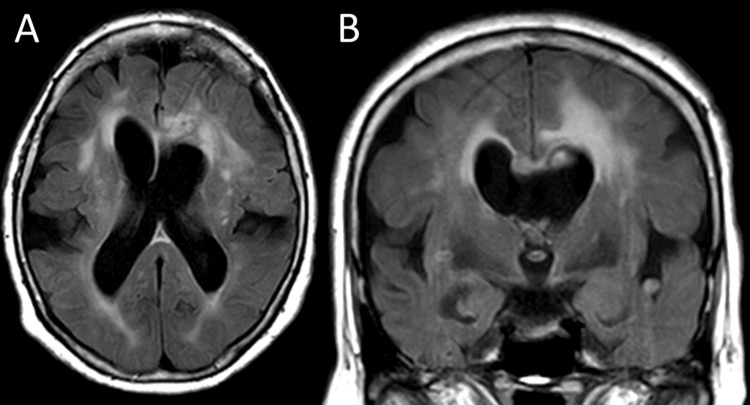
Postoperative MRI. The tumor at the septum pellucidum was removed (A, B).

Postoperative MRI revealed resection of the tumor, as in the intraoperative findings (Figure 3). The patient underwent ventriculoperitoneal shunting to address hydrocephalus and was transferred to another hospital for continued rehabilitation. She remained well 10 months after surgery.

## Discussion

IVPA is a relatively rare entity in children and even rarer in adults; thus, its epidemiology is unclear. The registry of the US National Cancer Institute over a 35-year period contained 86 cases of IVPA in patients aged 20 years and older (2.6% of all PAs) [2]. Previous studies reported 40 IVPAs in 38 patients over 20 years of age among seven case reports and six case series [3-15]. The most frequent sites for IVPA in adults were the lateral ventricle (57.5%) followed by the fourth ventricle (22.5%) and third ventricle (20.0%). Almost all lesions in the lateral ventricle were located unilaterally; only one case was located at the septum pellucidum, as in our case. The incidence of PA usually decreases with age, and IVPA in adults shows the same tendency, with a median age of 37.8 ± 12.7 years [3-15]. Two cases of IVPA have been reported in patients over 60 years of age [3]; therefore, our patient is the oldest reported case.

Relatively little is known about the behavior of PA in adults; therefore, the therapeutic strategy, in this case, was based on extrapolation of data from pediatric cases. However, recent studies have suggested aggressive tumor behavior in adults, with higher rates of progression, recurrence, and mortality than generally appreciated [1,2,16,17]. Additionally, high age at diagnosis is a risk factor for poor outcomes. Age older than 40 years is correlated with poor overall survival [1,3]. Johnson et al. revealed that survival rates declined significantly for each 10-year period with increasing age in adults, and the highest mortality rate was seen in the oldest age group: 60-month survival was 96.5% in patients under 19 years of age but 52.9% in patients over 60 years of age, excluding competing causes of death or misdiagnosis in older patients [2]. Furthermore, adult IVPA is associated with poorer overall- and progression-free survival compared with the general adult PA population [3]. Adult IVPA is also associated with a higher mortality risk, with a hazard ratio of 1.7, compared with tumors in the cerebellum in adult patients with PA [2].

One suspected factor associated with poor outcomes in adult IVPA is a low rate of gross total resection (GTR). A previous multivariable analysis revealed GTR as a significant positive prognostic indicator in adult IVPA [2,3]. The GTR rate in IVPA is the lowest at 22.0% for all PA locations [2]. IVPA is often associated with marked surgical challenges due to the proximity to critical and delicate functional and vascular brain structures. Although the existence of multiple lesions or cerebrospinal fluid dissemination is an additional factor that leads to residual tumor [4-7], it is still unclear which factors influence the development of leptomeningeal spread.

Although the poor prognosis associated with PA in older patients provides a rationale for adjuvant therapy after surgery, no standard chemotherapeutic regimen exists for adult PA, and adjuvant radiotherapy following resection remains controversial. To clarify the specific mechanisms of progression, dissemination, and recurrence or to evaluate adjuvant therapy in adult IVPA, additional pathological data, especially molecular information, is needed. PA is sometimes eas­ily diagnosed by classic histopathological features with hematoxylin and eosin staining. Many previous cases have been reported with or without limited histological examination. In particular, only minimal immunohistochemical and/or molecular studies have been performed and in some cases, patients are not referred for this testing. We identified seven adult IVPA cases that were histologically and molecularly documented in detail [3,6,8-10]. Some associated genetic abnormalities and their features in PA have been elucidated, but the findings remain controversial in adult IVPA. For instance, KIAA1549/BRAF fusion is common in cerebellar PA, but less common supratentorially and much less frequent in adult PA [18]. A case of supratentorial adult IVPA with confirmed KIAA1549/BRAF fusion [10] has been reported, but this was not reported in other cases. BRAF V600E mutations are more common in supratentorial PA [19] and rare in adults [17]. This genetic mutation has not been identified even in supratentorial adult IVPA [8]. Owing to the limited number of report­ed cases, the molecular biology of adult IVPA is still not well understood, and the different features between adults and children or between age groups in adults have not been clarified. If specific molecular alterations to acti­vate the MAPK pathway are detected in adult IVPA, the information will be beneficial for the prediction of patient outcomes or possible indications for molecular targeted therapy.

The risk of intraoperative bleeding in surgical intervention for IVPA was described previously; eight open surgical cases had > 200 ml blood loss [3,4]. IVPA is rare and difficult to diagnose on the basis of preoperative clinical and radiologic findings [3-5]; therefore, it should be considered in the differential diagnosis of intraventricular neoplasms. Referring to advances in endoscopic technology and instruments, Li et al. reported endoscopic surgery as an effective option for lateral ventricular tumors, with the advantage of less intraoperative bleeding compared with microscopic surgery [20]. Although endoscopic surgery for adult IVPA was performed in only five cases, and intraoperative blood loss in our patient was only 15 ml [3,8-10], no patients experienced excessive intraoperative bleeding.

## Conclusions

We described a unique case of PA that occurred as intraventricular multiple neoplasms in a patient with advanced age. Further studies on the pathological features of adult IVPA, including molecular studies, are needed to clarify the characteristics associated with aggressive behavior and elucidate new treatment indications or stratification. This diagnosis should be routinely considered in the differential diagnosis of intraventricular neoplasms showing uncommon findings as PA.
